# Machine learning predicts post-transplant muscle loss in hepatocellular carcinoma patients without sarcopenia

**DOI:** 10.1186/s12885-025-14973-5

**Published:** 2025-10-14

**Authors:** Jinyan Chen, Zhihang Hu, Huigang Li, Renyi Su, Zuyuan Lin, Jianyong Zhuo, Chiyu He, Ruijie Zhao, Wei Shen, Yajie You, Shuhan Jiang, Xuyong Wei, Shusen Zheng, Xiao Xu, Di Lu

**Affiliations:** 1 Institute of Translational Medicine, Zhejiang University School of Medicine, Hangzhou, 310058 China; 2 Department of Hepatobiliary and Pancreatic Surgery, Hangzhou First People’s Hospital, Hangzhou, 310006 China; 3 School of Basic Medical Sciences and Forensic Medicine, Hangzhou Medical College, Hangzhou, 310014 China; 4 NHC Key Laboratory of Combined Multi-organ Transplantation, Hangzhou, 310003 China; 5 Department of Hepatobiliary and Pancreatic Surgery, Shulan (Hangzhou) Hospital, Hangzhou, 310000 China; 6 Department of Hepatobiliary and Pancreatic Surgery, First Affiliated Hospital, Zhejiang University School of Medicine, Hangzhou, China; 7 School of Clinical Medicine, Hangzhou Medical College, Hangzhou, Zhejiang 310014 China; 8 Department of Hepatobiliary & Pancreatic Surgery and Minimally Invasive Surgery, Zhejiang Provincial People’s Hospital, Affiliated People’s Hospital, Hangzhou Medical College, 310014 Hangzhou, China

**Keywords:** Liver transplantation, Hepatocellular carcinoma, Sarcopenia, Muscle loss, Machine learning

## Abstract

**Objective:**

Developing a machine learning model to predict post-transplant muscle loss in hepatocellular carcinoma patients.

**Background:**

Liver transplantation is an effective treatment for selected HCC patients. However, severe muscle loss after liver transplantation is significantly associated with increased risk of mortality and recurrence. However, effective predictive methods remain inadequate.

**Methods:**

This study collected data from hepatocellular carcinoma patients who underwent liver transplantation over the past 2015 to 2020 at two hospitals. Propensity score matching and Cox regression analysis were conducted to establish muscle loss as an independent risk factor for recurrence. To construct the optimal predictive model for post-transplant muscle loss, we compared 50 machine learning models and use Recursive Feature Elimination to identify the most relative features.

**Results:**

Data from a total of 248 patients were collected. Kaplan-Meier analysis revealed a significant difference in prognosis between patients with and without sarcopenia before surgery. For patients without sarcopenia, postoperative muscle loss was identified as an independent risk factor for recurrence (HR = 2.38, *P* = 0.005). The best model was identified as the Imbalanced Random Forest, achieving an AUC of 0.832 on the non-sarcopenia cohort.

**Conclusions:**

A highly efficient model based on machine learning was developed to predict postoperative muscle loss in hepatocellular carcinoma patients undergoing liver transplantation, providing a valuable reference for the early detection of adverse events following the procedure.

**Supplementary Information:**

The online version contains supplementary material available at 10.1186/s12885-025-14973-5.

## Introduction

Hepatocellular carcinoma (HCC) ranks as the third leading cause of tumor-related mortality globally [1], and liver transplantation (LT) represents a radical treatment for selected HCC patients [2, 3].

Muscle loss after liver transplantation is an underappreciated but critical factor influencing patient recovery and long-term outcomes [4, 5]. Previous studies have highlighted the prognostic significance of sarcopenia in patients undergoing liver transplantation [5, 6], yet the dynamics of muscle loss in those without preoperative sarcopenia remain inadequately explored. This gap in understanding has impeded the development of effective predictive models, leaving clinicians without reliable tools to anticipate and mitigate this risk [5, 7].

In recent years, the advent of machine learning in healthcare has opened new avenues for predicting complex clinical outcomes [8]. By analyzing large datasets and identifying intricate patterns, machine learning models can offer superior predictive accuracy compared to traditional methods [9–11]. In this study, we aimed to harness these capabilities to develop a model capable of predicting muscle loss in HCC patients post-liver transplantation, with the potential not only to serve a clinical function but also to inspire further exploration in this field.

## Method

### Study population and data collection

**Fig. 1:**
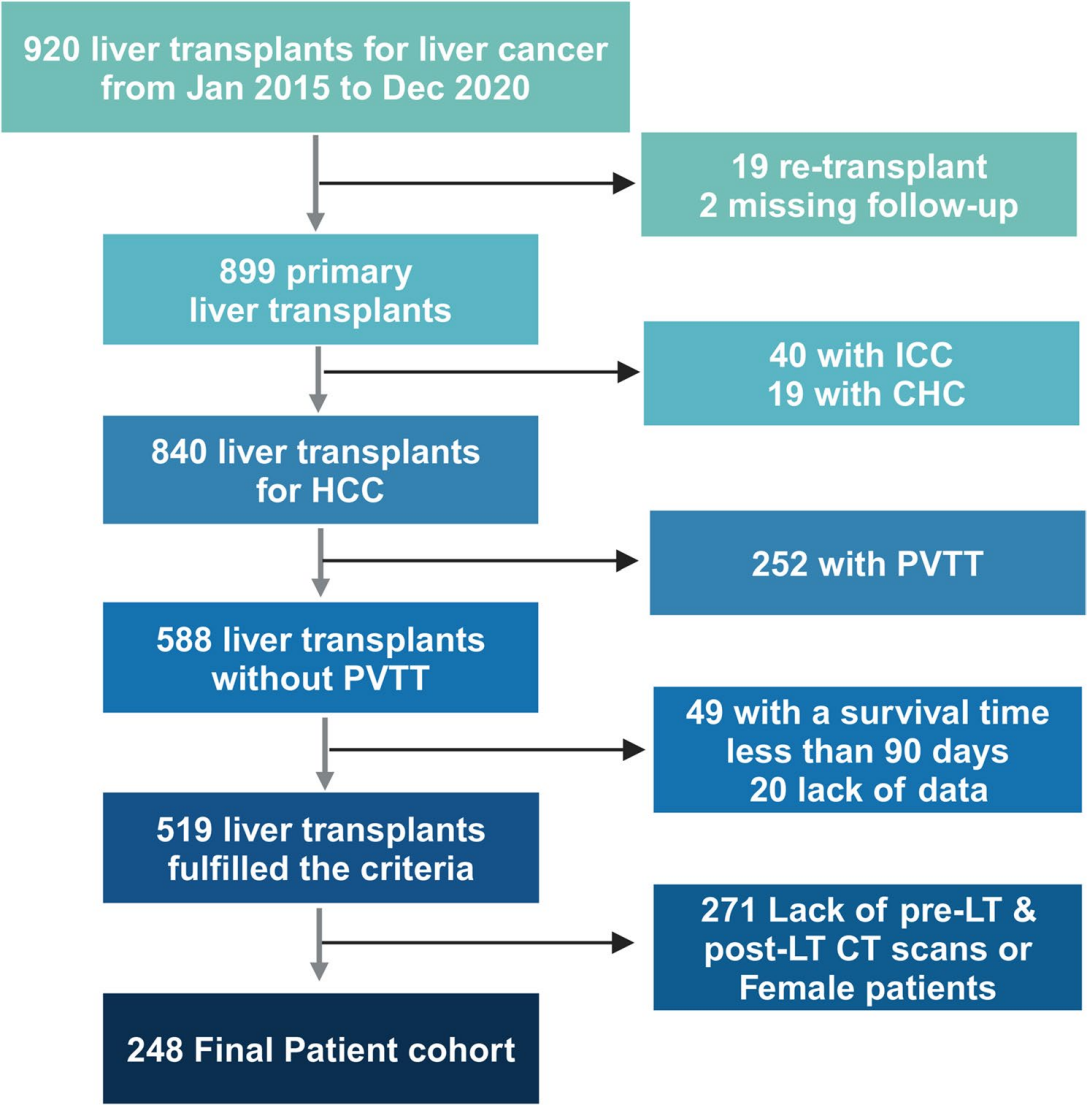
Flowchart of this study

This study included patients who underwent liver transplantation at the First Affiliated Hospital of Zhejiang University School of Medicine (Hangzhou, China) and Shulan (Hangzhou) Hospital (Hangzhou, China) between January 2015 and January 2021, with postoperative pathology confirming hepatocellular carcinoma (HCC). The flowchart depicting subject selection is presented in Fig. 1. Ultimately, we included 248 patients with complete preoperative and postoperative CT scans.

This research incorporated peripheral blood indicators, including complete blood count and liver function tests, collected within 2 weeks prior to surgery.

Post liver transplantation, recipients were followed up until December 31, 2022. During the follow-up period, recipients were managed according to standard protocols. Alpha-fetoprotein (AFP) levels were measured every 1–2 months [12]. Recipients underwent ultrasound, computed tomography (CT), or magnetic resonance imaging (MRI) examinations every 3–6 months after liver transplantation. Recurrence-free survival (RFS) was defined as the number of months from the date of surgery to the date of the first confirmed recurrence or death [13]. Metastatic sites of HCC were determined based on radiological evidence following standard HCC guidelines [12, 13]. The clinical and anthropometric parameters were collected from the China Liver Transplant Registry (CLTR) in strict accordance with the Regulations on Human Organ Transplantation and national legal requirements.

### Skeletal muscle assessment

Skeletal muscle was evaluated at the third lumbar vertebra (L3) of cross-sectional CT images using SliceOmatic software (version 5.0; Tomovision). The Hounsfield unit (HU) threshold of skeletal muscle was − 29 to + 150 HU as described previously [14]. The muscle area was standardized for the patient’s squared height in meters (m2) resulting in the skeletal muscle index (SMI). The measurement of body composition was conducted by two trained observers. Prior to the formal measurements, we randomly selected 50 CT images from the queue and provided them to the observers for assessing inter-observer reproducibility. One week later, we had the two observers repeat the same tasks to evaluate intra-observer reproducibility. The images of the aforementioned measurement results were validated by the anatomical radiologists. Before dynamic analyses, a precision test (30 CT images measured in duplicate by both observers) was performed to avoid random error caused by measurements according to a previously reported approach [15]. Once the precision error of the measurements is known, the magnitude of the change in muscle that indicates real biologic change can be determined by the least significant change (LSC). The value with the larger error in the results of the two observers was used in the subsequent discussion.

### Model development, feature selection and model explanation

Data from both institutions were combined and processed using a hybrid sampling technique, consisting of both oversampling and undersampling, combined with 5-fold cross-validation to avoid overfitting issues. This constitutes internal validation with pooled multi-institutional data rather than true external validation. Benchmark analysis with grid search was conducted among 50 machine learning models (Table S1) with 5-fold cross-validation to gain a stable model. AUC, Accuracy, F1, LogLoss, Precision, Recall were used to evaluate model performance.

The SHAP method (https://github.com/shap/shap) was adopted to gain the importance of features and explain model via R package “mlr3” [16]. Features selection was based on the strategy that sequential adding features from 1 to all features (46 clinical available indicators) consistent with importance rank. We selected the reduced-feature model due to its comparable AUC and enhanced clinical usability.

### Statistical analysis

Categorical variables were shown as numbers (percentages) and compared using the χ2 or Fisher exact test, as appropriate. Continuous variables were shown as medians (interquartile range) and compared using Kruskal-Wallis test. Comparisons of survival curves were examined via the Kaplan-Meier method with log-rank test. The optimal threshold for defining high muscle loss in the non-sarcopenia cohort was determined using the survminer package in R based on maximally selected log-rank statistics. As illustrated in Figure S1, this method plots the log-rank statistic for every possible cutoff point for muscle loss percentage. The value that corresponds to the maximum statistic (the peak of the curve) represents the optimal threshold that best separates patients into groups with different survival outcomes. In our cohort, this value was 16.8%, which was used to define high muscle loss. Propensity score-matching (PSM) analysis was performed using a 1:2 matching ratio with default nearest neighbor matching algorithm by “MatchIt” R package (version 4.5.5). P value < 0.05 was considered significant for analyses. All statistical analyses were performed by R language software (version 4.2.1.).

## Result

### Patient baseline characteristics

A total of 248 patients who underwent liver transplantation met the inclusion criteria of the study (Fig. 1). Patients with an SMI < 43.64 were classified as sarcopenic, yielding 69 cases (27.9%). In the non-sarcopenia cohort, we classified patients with postoperative muscle loss greater than 16.8% as having high muscle loss, encompassing 46 patients (25.7%). Baseline characteristics and surgical outcomes for the entire cohort and the propensity score-matched (PSM) cohort are presented in Table 1.

**Table 1:** Patient characteristics for entire cohort and PS-matched cohort

	Entire Cohort	Entire Cohort		*P* value	Non-sarcopenic Cohort		*P* value	PS-matched Non-sarcopenic cohort		*P* value
		Without Sarcopenia	Sarcopenia		Mild Muscle Loss	Severe Muscle Loss		Mild Muscle Loss	Severe Muscle Loss	
*n*	248	179	69		133	46		92	46	
Age (yr)	55.00 [48.00, 61.00]	55.00 [48.00, 61.00]	56.00 [51.00, 61.00]	0.407	55.00 [49.00, 61.00]	53.00 [46.00, 59.50]	0.163	56.00 [49.75, 61.00]	53.00 [46.00, 59.50]	0.1
BMI (median [IQR])	21.01 [20.05, 23.07]	21.45 [20.52, 23.44]	20.52 [19.38, 22.04]	<0.001	21.51 [20.76, 23.44]	21.26 [20.28, 23.29]	0.88	21.26 [20.76, 23.38]	21.26 [20.28, 23.29]	0.982
HBV Status	212 (85.5)	152 (84.9)	60 (87.0)	0.836	114 (85.7)	38 (82.6)	0.788	76 (82.6)	38 (82.6)	1
SMI(cm^2)	48.44 (8.37)	52.32 (6.05)	38.39 (4.08)	<0.001	51.81 (5.94)	53.79 (6.19)	0.055	53.57 (6.28)	53.79 (6.19)	0.844
MELD score	30.00 [15.75, 39.00]	30.00 [15.50, 39.00]	30.00 [16.00, 38.00]	0.994	29.00 [13.00, 39.00]	34.00 [21.00, 39.00]	0.073	30.00 [12.75, 39.00]	34.00 [21.00, 39.00]	0.151
Beyond Hangzhou criteria	34 (13.7)	17 ( 9.5)	17 (24.6)	0.004	13 ( 9.8)	4 ( 8.7)	1	7 ( 7.6)	4 ( 8.7)	1
AFP (μg/L)	19.35 [4.18, 143.82]	17.00 [4.05, 91.30]	36.70 [4.30, 506.70]	0.033	20.20 [4.00, 134.50]	11.57 [4.18, 52.10]	0.262	18.40 [3.65, 144.00]	11.57 [4.18, 52.10]	0.377
Tumor size (>5cm)	60 (24.2)	40 (22.3)	20 (29.0)	0.353	28 (21.1)	12 (26.1)	0.616	17 (18.5)	12 (26.1)	0.416
Total Tumor Diameter (>8cm)	89 (35.9)	64 (35.8)	25 (36.2)	1	47 (35.3)	17 (37.0)	0.985	29 (31.5)	17 (37.0)	0.655
Tumor number (>3)	48 (19.4)	31 (17.3)	17 (24.6)	0.259	21 (15.8)	10 (21.7)	0.488	12 (13.0)	10 (21.7)	0.285
Differentiation (Poor)	62 (25.0)	40 (22.3)	22 (31.9)	0.164	31 (23.3)	9 (19.6)	0.749	22 (23.9)	9 (19.6)	0.718
Microvascular invasion	100 (40.3)	65 (36.3)	35 (50.7)	0.054	47 (35.3)	18 (39.1)	0.777	31 (33.7)	18 (39.1)	0.66
Deceased	67 (27.0)	39 (21.8)	28 (40.6)	0.005	23 (17.3)	16 (34.8)	0.023	11 (12.0)	16 (34.8)	0.003
OS (days)	1,170.50 [760.50, 1,463.50]	1,198.00 [830.50, 1,465.50]	957.00 [538.00, 1,445.00]	0.015	1,259.00 [900.00, 1,525.00]	985.00 [749.00, 1,382.50]	0.033	1,282.50 [960.75, 1,525.75]	985.00 [749.00, 1,382.50]	0.012
Recurrence	81 (32.7)	46 (25.7)	35 (50.7)	<0.001	28 (21.1)	18 (39.1)	0.026	19 (20.7)	18 (39.1)	0.035
RFS (days)	1,150.00 [693.75, 1,463.50]	1,195.00 [750.50, 1,465.50]	924.00 [331.00, 1,445.00]	0.029	1,232.00 [834.00, 1,525.00]	928.50 [556.25, 1,382.50]	0.024	1,282.50 [946.00, 1,525.75]	928.50 [556.25, 1,382.50]	0.007
Liver metastasis	46 (18.5)	26 (14.5)	20 (29.0)	0.015	13 ( 9.8)	13 (28.3)	0.005	8 ( 8.7)	13 (28.3)	0.006
Lung metastasis	48 (19.4)	25 (14.0)	23 (33.3)	0.001	15 (11.3)	10 (21.7)	0.129	12 (13.0)	10 (21.7)	0.285
Bone metastasis	20 ( 8.1)	15 ( 8.4)	5 ( 7.2)	0.973	9 ( 6.8)	6 (13.0)	0.31	4 ( 4.3)	6 (13.0)	0.131
Brain metastasis	6 ( 2.4)	3 ( 1.7)	3 ( 4.3)	0.444	3 ( 2.3)	0 ( 0.0)	0.718	1 ( 1.1)	0 ( 0.0)	1

**Fig. 2:**
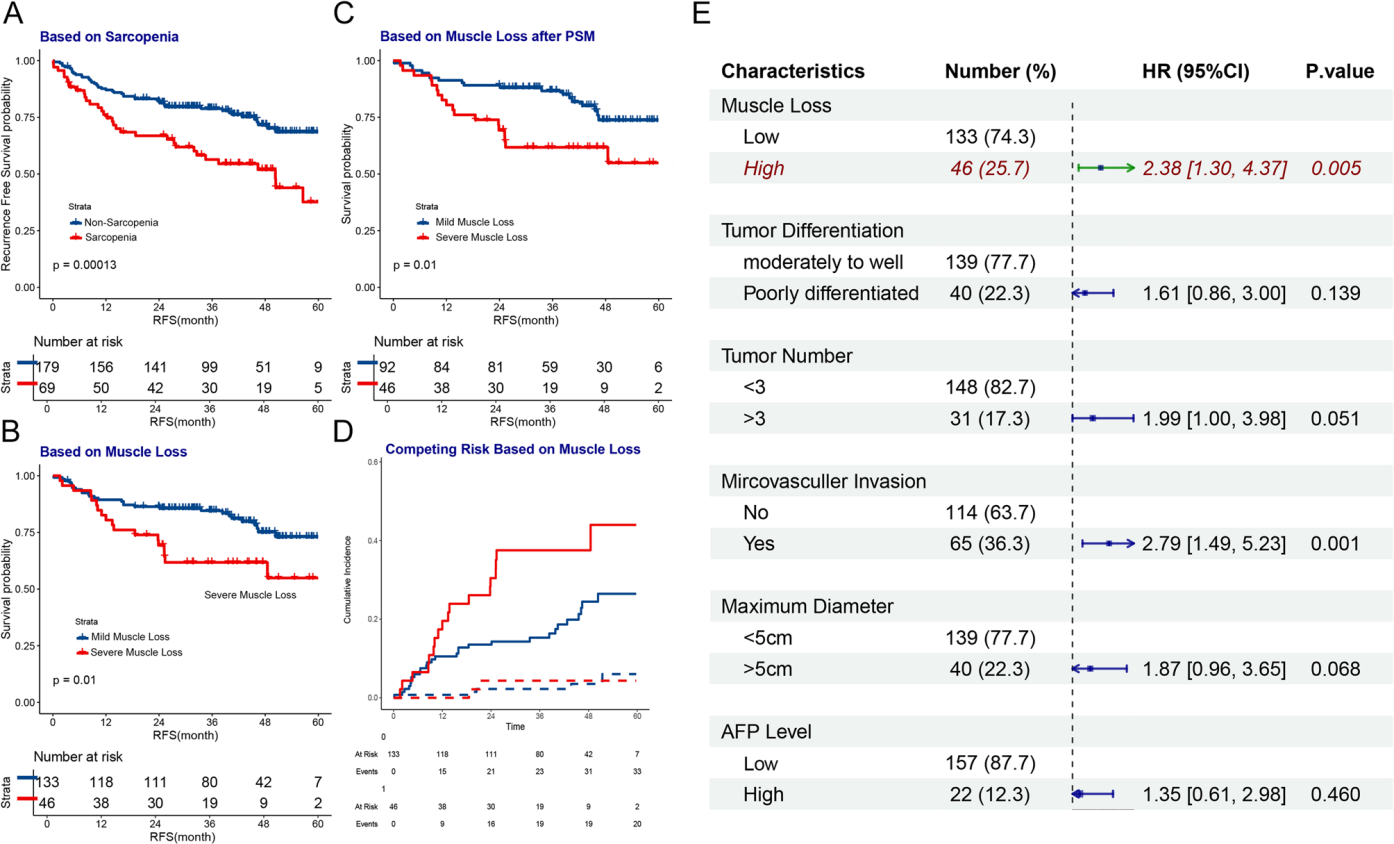
**A** Kaplan-Meier survival curve shows the recurrence-free survival rates of the preoperative sarcopenia and non-sarcopenia groups in this cohort. **B** Kaplan-Meier survival curve shows the recurrence-free survival rates of the high muscle loss and low muscle loss groups within the preoperative non-sarcopenia cohort. **C** Kaplan-Meier survival curve shows the recurrence-free survival rates of the high muscle loss and low muscle loss groups within the preoperative non-sarcopenia cohort after propensity score matching. **D** Competing risk analysis of the high muscle loss and low muscle loss groups within the the preoperative non-sarcopenia cohort. **E** Forest plot of the multivariate Cox regression analysis in the non-sarcopenia group

Kaplan-Meier analysis demonstrated that patients with sarcopenia had a poorer prognosis (Fig. 2A). Patients with high muscle loss had worse prognosis, and this trend persisted after 1:2 PSM (Fig. 2C, *P* = 0.01). Competing risk analysis demonstrated that the differences in RFS due to muscle loss were attributable to recurrence, rather than non-recurrence-related mortality (Fig. 2D). Subsequently, we incorporated the following variables—age, BMI, HBV status, muscle loss, SMI, MELD score, AFP levels (> 400 µg/L), tumor differentiation, tumor number, microvascular invasion, and maximum tumor diameter—into a univariate Cox analysis, followed by multivariate Cox analysis, which included factors below, degree of muscle loss, tumor differentiation, number of tumors, microvascular invasion, maximum tumor diameter, and AFP levels(Fig. 2E). Our findings revealed that high muscle loss is an independent risk factor for recurrence of liver transplant recipients with hepatocellular carcinoma (HR = 2.38, *P* = 0.005). However, there is still a lack of effective preoperative models in clinical practice to predict the extent of postoperative muscle loss. To address this, we embarked on the development of such a model.

### Model development

**Fig. 3:**
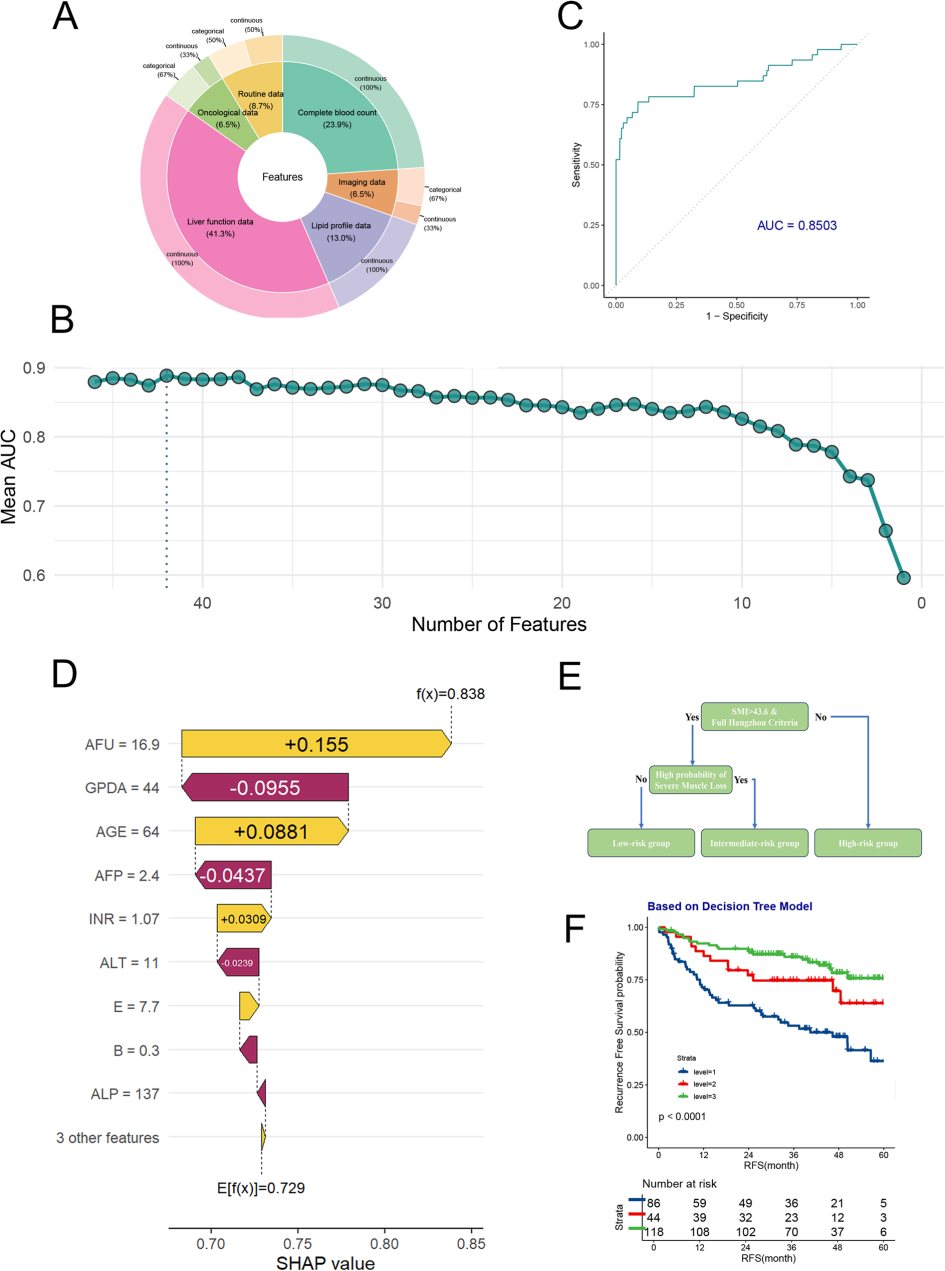
**A** Overview of the 46 features included in the development of the machine learning model. **B** AUC curve during the recursive feature elimination process. **C** ROC curve of the model in the preoperative non-sarcopenia group. **D** Waterfall plots of the model, showing the contribution of each variable to the model’s predictions. **E** Schematic diagram of the decision tree model. **F** Kaplan-Meier survival curve shows the recurrence-free survival rates of the high-risk, intermediate-risk, and low-risk groups based on the decision tree model

To develop a machine learning model for predicting postoperative muscle loss in liver transplant patients using clinical indicators, we collected 46 clinical features from the patients (Fig. 3A). The features included in the analysis were age, height, BMI, HBV infection status, MELD score, AFP, tumor differentiation, tumor number, maximum tumor diameter, total tumor size, microvascular invasion, albumin, albumin-to-globulin ratio, ALT, AST, INR, monocyte count, neutrophil count, platelet count, SMI, white blood cell count, lymphocyte count, eosinophil count, basophil count, hemoglobin concentration, red blood cell count, RDW-MHV, MCHC, HGB, PCT, GGT, ALP, indirect bilirubin, direct bilirubin, ChE, TBA, GPDA, AFU, ADA, TG, TC, HDL, LDL, VLDL, and fasting blood glucose. We then compared the performance of 50 different machine learning models using 5-fold cross-validation. Among these, the Imbalanced Random Forest demonstrated the best performance for this clinical issue (Table S1). This model is specifically designed to handle imbalanced outcome data, making it well-suited for our clinical problem.

To streamline the model’s input features, we employed Recursive Feature Elimination (RFE) to systematically reduce the number of features from 46 down to 1. We found that including 12 features resulted in comparable performance to the model that used all features (Fig. 3B). As a result, we incorporated these 12 indicators—AFU, GPDA, AGE, AFP, INR, ALT, E, B, ALP, ADA, GGT, and BMI—into the final model. The model achieved an AUC of 0.8503(Fig. 3C).

We used the SHAP method to interpret this machine learning model, providing insights for future research. Among the final 12 clinical features, the top 4 important features related to muscle loss, as ranked by SHAP values, were AFU, GPDA, AGE, and AFP.

### Decision tree model incorporating Hangzhou criteria effectively stratifies patient prognosis

The 12 features included in the model are all preoperative peripheral blood characteristics. To use simple preoperative blood tests and the preoperative sarcopenia status to stratify patient prognosis, we combined the predicted probabilities from the model with the Hangzhou criteria to develop a straightforward decision tree model (Fig. 3E). This decision tree model effectively stratifies patient prognosis (Fig. 3F).

**Fig. 4:**
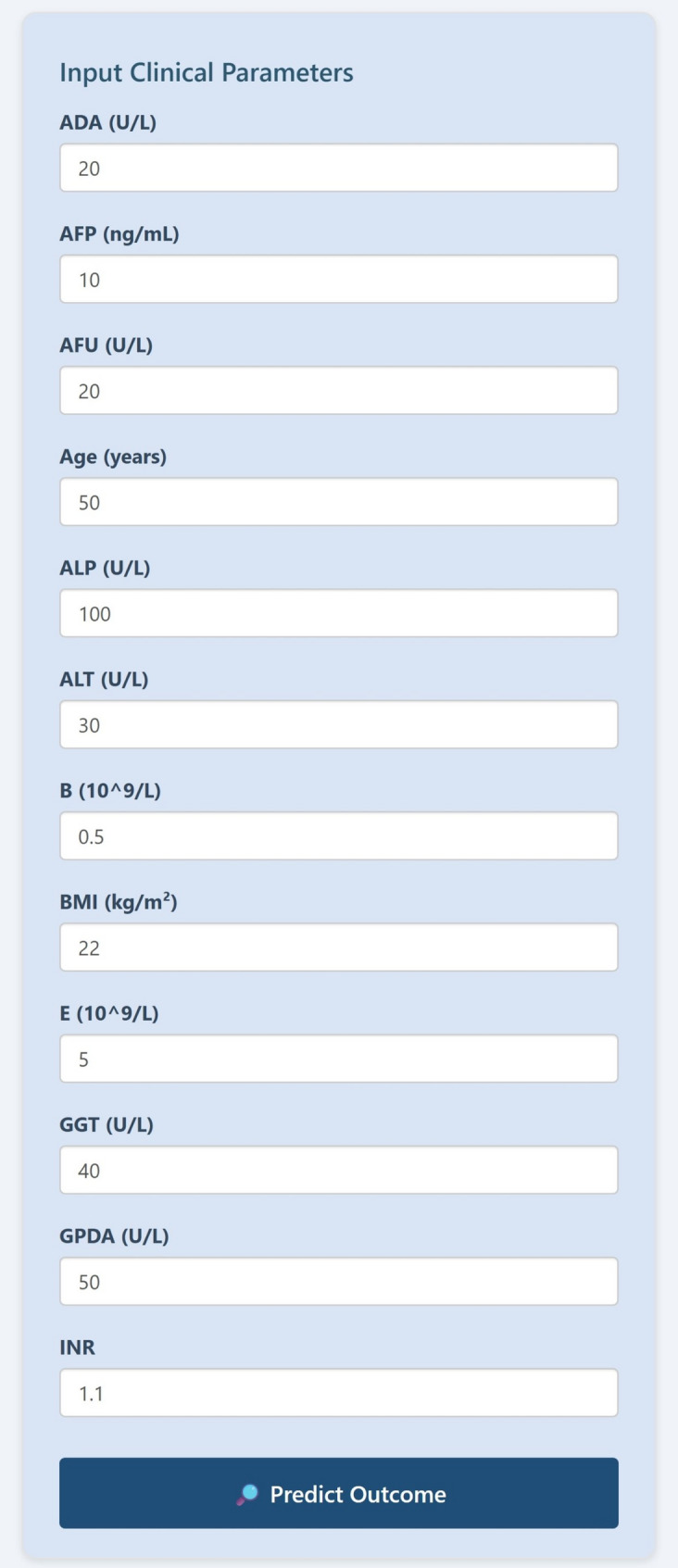
Convenient web application for prediction of Muscle loss after liver transplantation in clinical modality. Please note: To comply with institutional data security and patient privacy policies, this prediction tool is currently deployed on an internal server for validation and is not publicly accessible at this time

The 1-, 3-, and 5-year recurrence-free survival rates for high-risk patients were 71.5%, 53.2%, and 36.3%, respectively. For intermediate-risk patients, these rates were 86.4%, 74.6%, and 63.9%, respectively. For low-risk patients, the 1-, 3-, and 5-year recurrence-free survival rates were 92.3%, 85.9%, and 75.7%, respectively (*p* < 0.0001). This decision tree model is both easy to use and clinically effective. To facilitate clinical implementation, we developed a user-friendly web-based prediction tool using R Shiny framework (Fig. 4). This interactive application allows clinicians to input the 12 required clinical variables through an intuitive interface and immediately receive the predicted probability of severe post-transplant muscle loss, along with risk stratification categories. The web application demonstrates how the model could be seamlessly integrated into existing clinical decision-making processes during pre-transplant evaluation, providing immediate risk assessment to guide clinical management decisions.

## Discussion

In this study, we developed a machine learning model to predict postoperative muscle loss in liver transplant recipients, with a particular focus on patients without preoperative sarcopenia. Our findings underscore the significant impact of muscle loss on post-transplant outcomes, highlighting the need for early identification and targeted interventions.

Muscle loss, a common yet often underrecognized complication after liver transplantation, has profound implications for patient prognosis [5]. Previous studies have established sarcopenia as a predictor of poor outcomes in liver transplant recipients [7, 17, 18]. The use of machine learning in our study allowed us to identify key clinical features that predict muscle loss with high accuracy. Notably, the final model included 12 preoperative peripheral blood features, which are easily accessible in clinical settings. By integrating these features into a decision tree model, we developed a tool that not only predicts muscle loss but also stratifies patients into different risk categories for recurrence-free survival. This stratification is crucial for guiding postoperative management, particularly in tailoring follow-up and intervention strategies to individual patient risk profiles.

The machine learning model identified several interesting biomarkers as key predictors of muscle loss, with AFU and GPDA emerging as the most important features. AFU, a lysosomal enzyme involved in glycoprotein degradation, may reflect altered cellular metabolism and protein turnover that predisposes to muscle catabolism [19–21]. GPDA, which plays a crucial role in cellular energy metabolism, may indicate underlying metabolic dysfunction that impairs muscle maintenance during post-transplant recovery [22–24]. More specifically, altered GPDA activity could reflect mitochondrial dysfunction in skeletal muscle, where efficient energy production through the glycerol-phosphate shuttle is critical for muscle protein synthesis. Age represents a well-established risk factor, as aging is associated with progressive decline in muscle protein synthesis rates and increased susceptibility to catabolic stimuli. AFP elevation may indicate more aggressive tumor biology and associated systemic inflammation, both promoting muscle-wasting cytokine release [7]. INR reflects hepatic synthetic function and coagulation status, with elevated values suggesting impaired protein synthesis capacity that could compromise muscle protein maintenance [14]. ALT elevation indicates hepatocellular injury and may reflect altered amino acid metabolism crucial for muscle protein synthesis. Eosinophil and basophil counts may reflect underlying inflammatory or allergic responses that could promote muscle catabolism through inflammatory mediator release. ALP elevation could indicate increased bone turnover and tissue remodeling, suggesting accelerated catabolic processes that may extend to muscle tissue [7, 21]. ADA plays a role in purine metabolism and immune function, with altered levels potentially reflecting metabolic dysfunction affecting muscle energy metabolism [24]. GGT reflects oxidative stress and glutathione metabolism, with elevation indicating increased oxidative burden that can directly damage muscle proteins and impair muscle maintenance. BMI represents baseline nutritional status and metabolic reserve, with lower values indicating reduced capacity to maintain muscle mass during post-transplant metabolic stress. While the precise mechanistic pathways linking these biomarkers to muscle loss require further investigation, their identification through our machine learning approach suggests novel biological relationships that warrant future mechanistic studies and may represent previously unrecognized metabolic signatures of muscle loss risk.

The decision tree model developed in this study has potential clinical applicability due to its reliance on routine preoperative laboratory parameters that are already collected during standard pre-transplant evaluation. Clinicians could potentially integrate this tool into existing workflows to identify patients at high risk for post-transplant muscle loss. However, this study provides no data on the effectiveness of any interventions for preventing muscle loss. Targeted interventions such as prehabilitation programs, intensive nutritional support, and enhanced monitoring protocols remain hypothetical and unproven until formally evaluated in controlled studies. We emphasize that this study provides no evidence supporting the effectiveness of any interventions in preventing muscle loss or improving outcomes. The potential effectiveness of interventions in altering outcomes remains completely unknown and requires prospective validation, which represents an important direction for future research. The model’s primary value lies in risk identification; any clinical benefit of subsequent interventions remains unproven and requires validation in controlled studies.

Future research should focus on validating these findings in larger cohorts and conducting controlled studies to determine whether any interventions can effectively mitigate muscle loss and enhance recovery in liver transplant patients. Currently, no evidence exists supporting the effectiveness of nutritional or physical interventions in this specific population.

This study has several limitations that should be acknowledged. We conducted multi-institutional internal validation by pooling data from two centers and applying 5-fold cross-validation, rather than using one center for training and another for independent external validation. Although both institutions are located within the same geographic region and may share latent biases including similar clinical protocols, data infrastructure, and regional healthcare practices, true external validation would require using completely separate datasets where one institution’s data trains the model and geographically/administratively independent cohorts test it. The rigorous 5-fold cross-validation employed in our study is a well-established method for assessing generalizability and provides reliable estimates of out-of-sample performance, which substantially mitigates concerns about overfitting and enhances confidence in the model’s potential transferability.

The retrospective nature of our study inevitably introduces selection bias when assigning patients to different groups. To mitigate this, we utilized propensity score matching to balance known influential confounding factors. Nonetheless, the study remains subject to the impact of unidentified confounding variables. The relatively small sample size prompted us to use rigorous cross-validation to optimize model parameters and training.

While our study focused specifically on hepatocellular carcinoma patients, this approach was deliberate to ensure a homogeneous patient population and reduce confounding variables associated with different underlying liver diseases. Despite the relatively modest sample size, our rigorous methodology including 5-fold cross-validation, multi-center data collection, and comprehensive algorithm comparison enhances the robustness of our findings. Future studies expanding to other liver transplant indications and larger cohorts will be essential for broader clinical implementation, though our current work provides a solid foundation for predictive modeling in this important patient population.

Despite these limitations, our study demonstrates several strengths that support the potential generalizability of our findings. The model’s reliance on standardized, universally available clinical parameters, combined with its consistent performance across two different institutions, suggests robust predictive patterns that are not institution-specific.

We plan future external validation using independent cohorts from other regions, testing models on entirely separate datasets. Our current multi-institutional internal validation provides a foundation, but external validation is essential before clinical implementation.

## Supplementary Information

Supplementary Material 1.

Supplementary Material 2.

## Data Availability

The data are available from the corresponding author upon request.

## Abbreviations

HCC: Hepatocellular carcinoma
AFU: α-L-Fucosidase
AFP: Alpha-Fetoprotein
GPDA: Glycerol-3-Phosphate Dehydrogenase Activity
ALT: Alanine Aminotransferase
ALP: Alkaline Phosphatase
E: Eosinophil
B: Basophil
ROC: Receiver Operating Characteristic
AUC: Area under the time-dependent receiver operating characteristic curve
HR: Hazard Ratio
CI: Confidence interval
