# Supplementary figures and images for: Machine learning predicts post-transplant muscle loss in hepatocellular carcinoma patients without sarcopenia
Source: BMC Cancer. 2025 Oct 14;25:1565. doi: 10.1186/s12885-025-14973-5 (PMC12522249; doi:10.1186/s12885-025-14973-5)

SMI Cutpoint Optimization Process  
Maximum statistic = 2.476 at cutpoint = 0.168

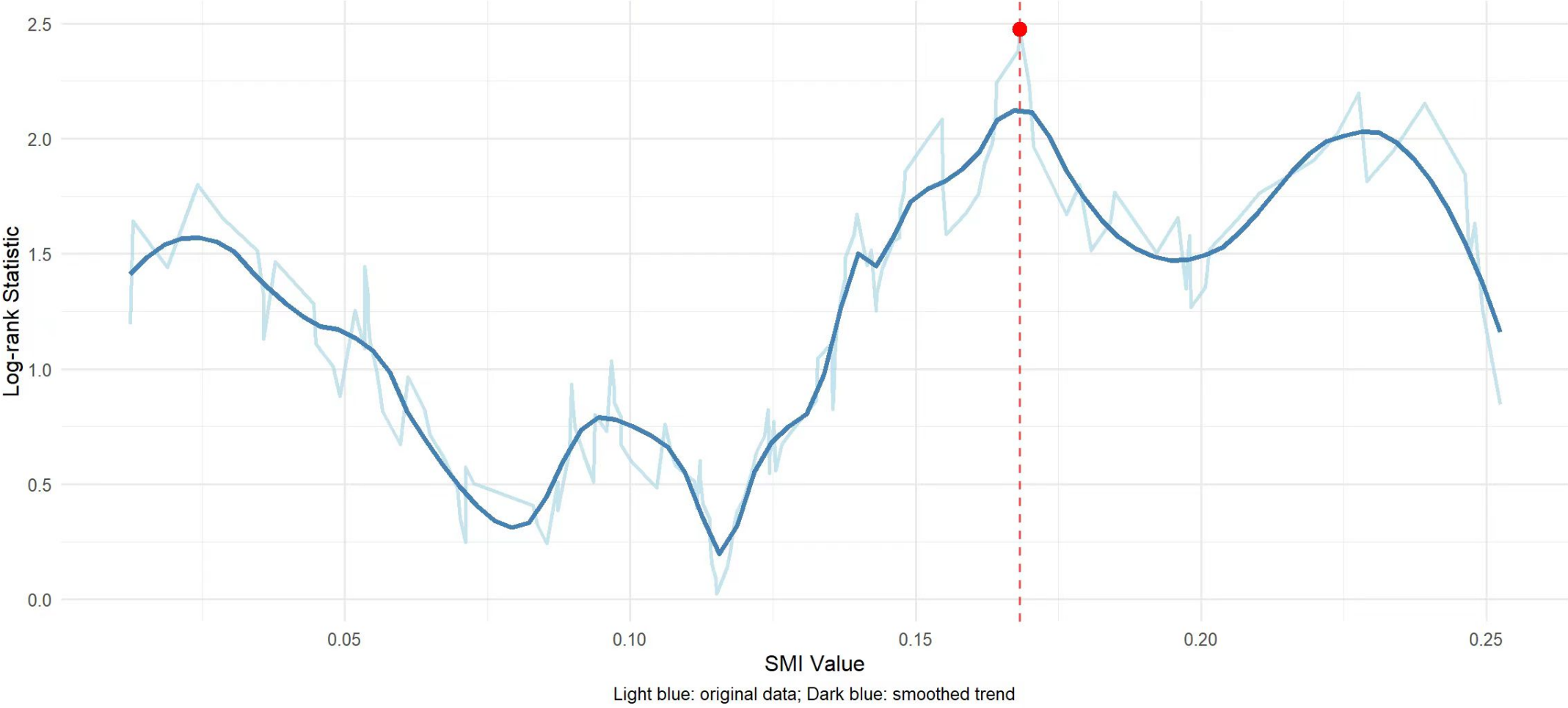

Supplement: Supplementary file 2 — Supplementary Material 2. [file 12885_2025_14973_MOESM2_ESM.pdf]
